# Dynamic apparent transition resistance data in spot welding of aluminized 22MnB5

**DOI:** 10.1016/j.dib.2016.06.063

**Published:** 2016-07-05

**Authors:** Jonny Kaars, Peter Mayr, Kurt Koppe

**Affiliations:** a Chair of Welding Engineering, Technische Universitaet Chemnitz, 09126 Chemnitz, Germany; b Chair of Production Engineering, Anhalt University of Applied Sciences, 06366 Koethen, Germany

**Keywords:** Spot welding, Transition resistance, 22MnB5, Simulation

## Abstract

In-situ resistance measurements of aluminized 22MnB5 steel using a current ramp of 500 A/ms at welding force levels from 2 kN to 8 kN were conducted to obtain data on the dynamic resistance behaviour in spot welding of the material for varying mechanical and electrical loads. The data has been successfully used to calibrate a numerical transition resistance model (KMK-model, Kaars et al., 2016 [1]) in Kaars et al. (2016) [2].

**Specifications Table**

**Table:** 

Subject area	*Engineering*
More specific subject area	*Spot Welding*
Type of data	*Graph, Table*
How data was acquired	*In-situ measurements with a welding machine*
Data format	*Raw, analyzed*
Experimental factors	*Untempered sheets of 1.5 mm thickness with AlSi-coating AS150 were heat treated in a furnace at 940* *°C for 360* *s, thereafter immediately quenched in a press. Final specimen geometry was fabricated by water jet cutting.*
Experimental features	*Resistance measurements were carried out by applying a welding current ramp on two sheets of the material in a XC-type spot welding gun. A welding current sloped with 500 A/ms up to 12 kA was used; the welding force F was varied in the range of 2–8 kN.*
Data source location	*Technische Universitaet Chemnitz, Germany*
Data accessibility	*Data is within article*

**Value of the data**

• The measured data gives insight in the electric resistance of aluminium-silicon coated 22MnB5 steel for a variety of mechanical and electrical contact loads, measured under the same experimental conditions, and therefore comprehensively characterizes the materials electric behaviour. Available data in literature only gave exemplary information on the transition resistance of aluminized 22MnB5 for varying, exemplary conditions.
• The data sets provided are suited to compare the resistance properties of different coatings or heat treatments to those of the coating investigated, and therefore to estimate the changed heat contribution of the coatings resistivity to the total welding heat. Due to lack of data available, the quantity of the effect of the sheet metals heat treatment on its resistance yet remains unclear.
• For numerical welding simulation, the data can be used to further enhance existing and develop new numerical models describing the specific dynamic transition resistance of the material [1], [2] The raw data can be used to define tabular resistance models as well. Suitable data to do so has not been published for aluminized 22MnB5 yet.

## 1 Data

**Fig. 1:**
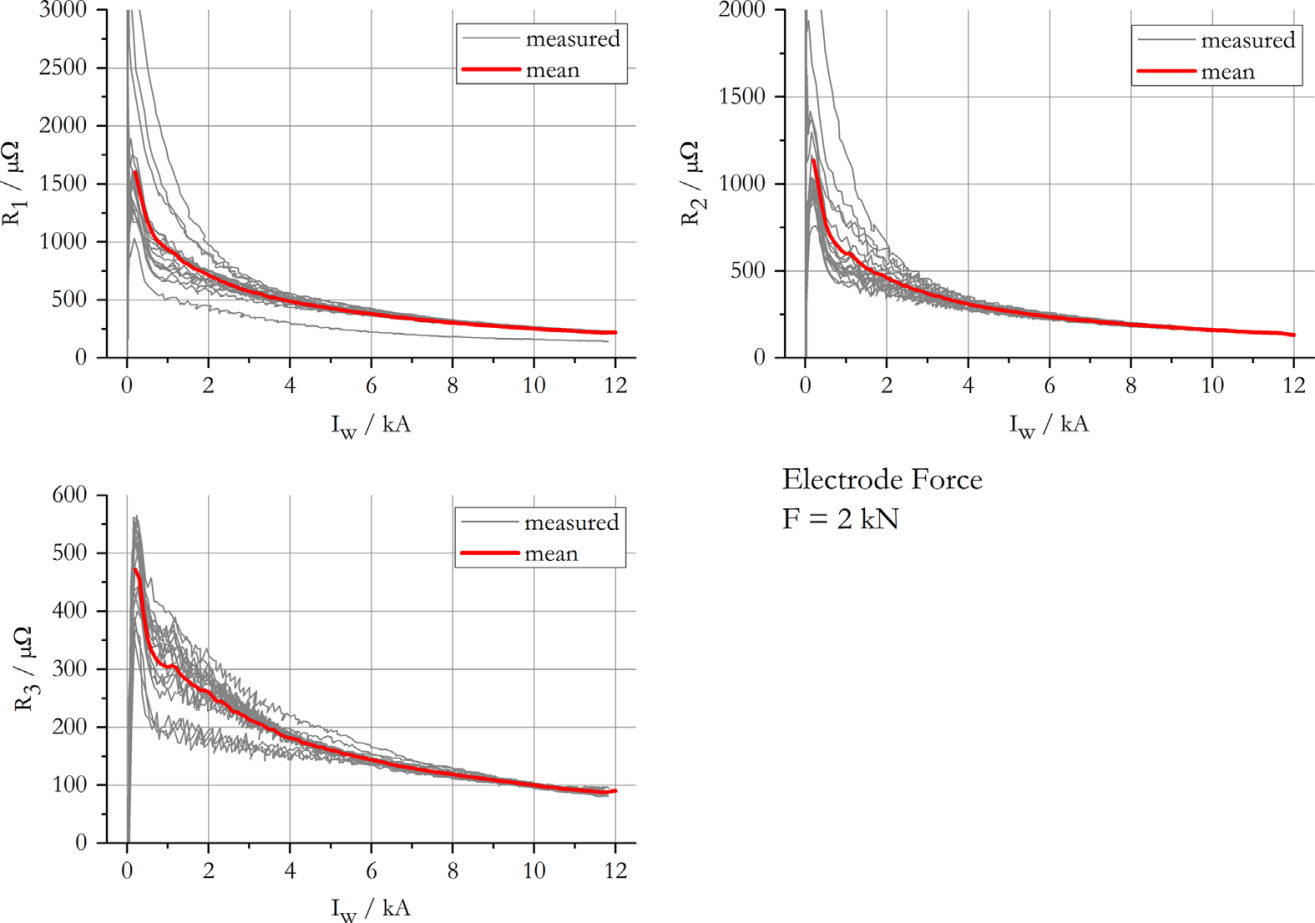
Measured and mean resistance curves at an electrode force of 2 kN.

**Fig. 2:**
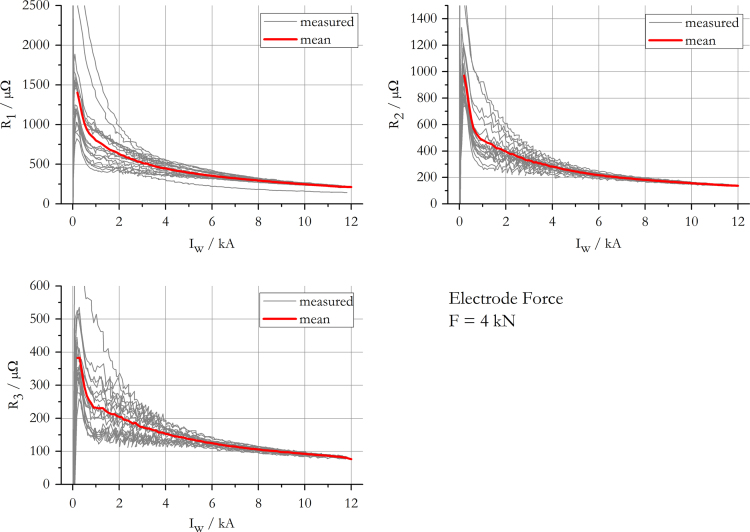
Measured and mean resistance curves at an electrode force of 4 kN.

**Fig. 3:**
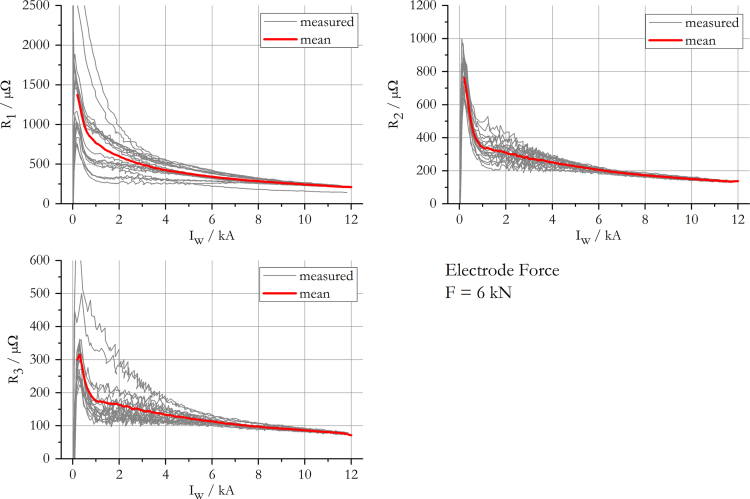
Measured and mean resistance curves at an electrode force of 6 kN.

**Fig. 4:**
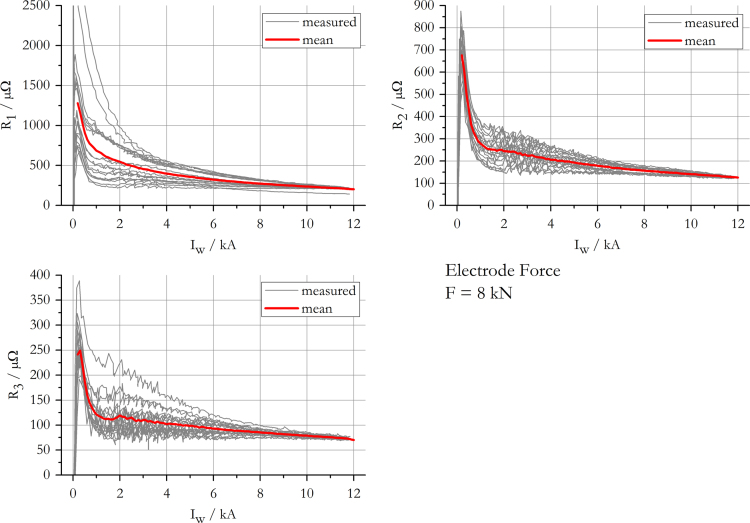
Measured and mean resistance curves at an electrode force of 8 kN.

The data presented in this work gives information on the apparent transition resistance [3] in spot welding of 22MnB5+AS150 and its standard deviation, while a monotonously increasing current is applied. The results include data for electrode forces of 2 kN, 4 kN, 6 kN and 8 kN and are summarised in Fig. 1, Fig. 2, Fig. 3, Fig. 4. The mean resistance as a function of electric current for each force level, along with the standard deviation, is appended in Supplementary Table 1.

## 2 Experimental design, materials and methods

The investigated material was delivered as ferritic-perlitic sheets of 1.5 mm thickness with an Aluminium-Silicon-coating of 150 g/m². After a heat treatment for 360 s at 940 °C in a lab furnace the sheets were manually transferred in less than 6 s in a press in order to quench the sheet and receive the martensitic structure of the base material as it is used in industrial applications with an average tensile strength of $R_{m}=1481\pm 53\;\mathrm{MPa}$ as determined by tensile tests. During the heat treatment, the Aluminium-Silicon-coating transformed into a mostly intermetallic surface layer with a characteristically fine roughness and brittle structure [4], [5]. Subsequent electron dispersive X-ray analysis determined the coating to consist of 17 µm of diffusion zone with an intermetallic aluminium-iron layer of 23 µm on top.

**Fig. 5:**
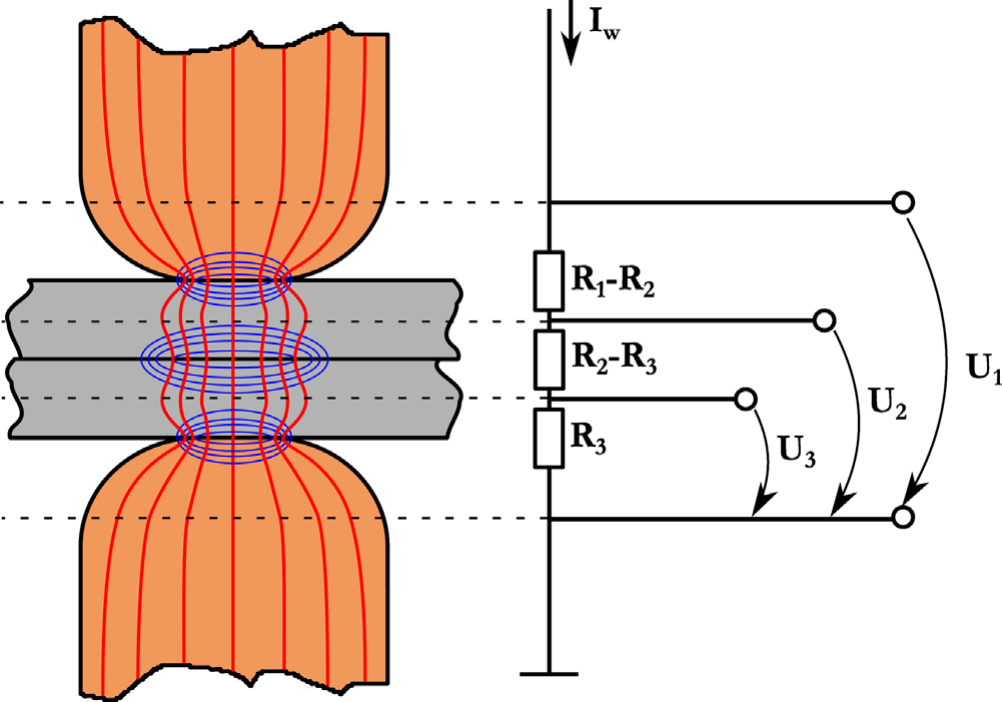
Definition and location of potential taps and apparent transition resistances; red: current paths, blue: lines of equal potential.

The resistance measurements were carried out using a XC-type spot welding gun [6] equipped with electrode tips of the type ISO 5821-B0-16-20-40-6-45, which transferred the welding current ramp $I_{w}$ of 500 A/ms up to 12 kA on the two sheets between the electrodes. The welding power source was an inverter power source with programmable current control. The measuring lines were soldered to the electrode cap holders, the connection to the sheets was established by means of strong clamps. Voltage and current were recorded with a sampling rate of 10 kHz. Each voltage $U_{i}$ was measured independently, one at a time. Within each force level, the measurements were repeatedly carried out in the sequence $U_{1}-U_{2}-U_{3}$ up to six datasets per test point. Then the electrodes were milled. The described measuring sequence was then repeated another two times, resulting in a total of eighteen datasets per test point. The described method was selected to ensure an even distribution of potential electrode wear and other influences in the results. Fig. 5 gives an overview over the experimental setup.

Afterwards, the corresponding resistance was computed from the voltage curve by using the relation:

$$R_{i}\left(I_{w}\right)=\frac{U_{i}\left(t\right)}{I_{w}\left(t\right)}\;\{i\in N|1\leq i\leq 3\}\wedge t=f(I_{w})$$

The resulting resistance data was then compressed by computing the mean curve and its standard deviation, both as a function of the electric current.
